# The Pole Term in Linear Response Theory: An Example From the Transverse Response of the Electron Gas

**DOI:** 10.6028/jres.113.023

**Published:** 2008-10-01

**Authors:** Zachary H. Levine, Eric Cockayne

**Affiliations:** National Institute of Standards and Technology, Gaithersburg, MD 20899

**Keywords:** electron gas, *f*-sum rule, Lindhard dielectric function, linear response theory, second order pole, transverse dielectric function

## Abstract

In linear response theory, the dielectric response at zero frequency sometimes appears to violate the *f*-sum rule, which has apparent implications for causality. Here, we study the origin of this apparent discrepancy, focusing on Lindhard’s formula for the transverse response of the electron gas. At non-zero frequency, first-order poles contribute to the imaginary part of the dielectric function in the usual way. At zero frequency, second-order poles contribute in a way which forces a careful consideration of the notation of summation and integration to avoid an error. A compact formula for the contribution of the second-order poles is presented. The sense in which the *f*-sum rule is satisfied is discussed.

## 1. The *f*-Sum Rule and Transverse Response

Causality—the principle that the state of a system depends upon its past but not its future—is expressed in the frequency domain as the principle that a causal response function has no poles in the closed upper half plane. Cauchy’s theorem may then be used to derive the relationship between the real and imaginary parts of such a function, known in physics as the Kramers-Kronig relation. The dielectric function *ε* (*ω*) is an example of such a function.

Since *ε* (*ω*) has no poles in the closed upper half plane, the same is true of [*ε* (*ω*) − 1]*ω*. Cauchy’s theorem implies that

**Fig. 1:**
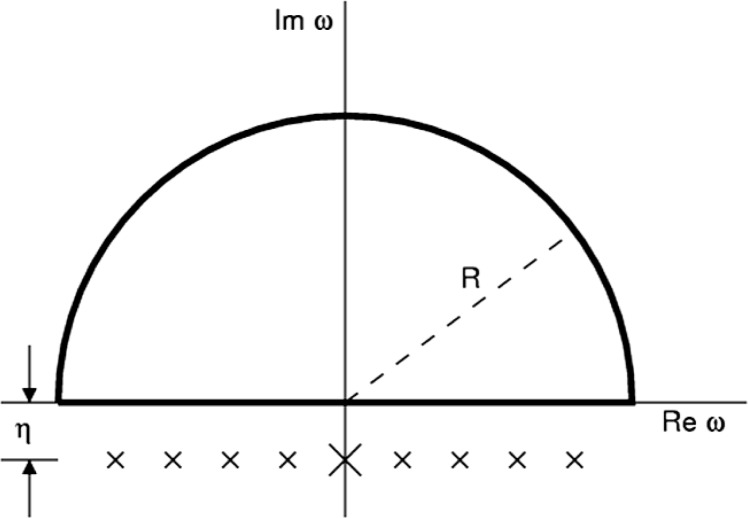
The contour of integration used to derive the *f*-sum rule. The limit of large radius, i.e., *R* → ∞ will be taken. The contour of integration may be taken to be exactly on the real axis because technically, for any finite *η*, the poles are in the lower half plane. The first-order poles at finite frequencies are shown with a small ×. These arise from the fourth term in Eq. (9). The second-order pole at zero frequency is shown with a large ×. Contributions to the second order pole arise from the second and third terms in Eq. (9). Note that these have the same small imaginary part, namely *η*, which is related to the rate constant in the exponential which controls how fast the electric fields are turned on from a vanishingly small value in the distant past.

0=Im∮dω[ε(ω)−1]ω (1)

along the standard contour given in Fig. 1. For a system of electrons in an electromagnetic field, the high-frequency response is given by the free electron form

$$\varepsilon (\omega )=1-\frac{\omega_{p}^{2}}{\omega^{2}}+O(\omega^{-4})$$ (2)

where *ωp* is the plasma frequency given by $\omega_{p}^{2}=4\pi ne^{2}/m$, where *n* is the number density of electrons, *m* is the electron mass, and −*e* is the charge on the electron [1–3]. The *O*() notation describes the limiting behavior [4]. We may break the contour integral into two parts and use Eq. (2) to simplify the integration along the semicircle. Defining *ω* = *Re^iθ^*,

0=limR→∞∫−RRdωImε(ω)−∫0πdθReiθωp2R2e2iθReiθ+O(R−2). (3)

The result

πωp2=∫−∞∞dωImε(ω)ε (4)

is known variously as the *f*-sum rule, the oscillator-strength sum rule, and the Thomas-Reiche-Kuhn sum rule. The *f*-sum rule may also be regarded as a high-frequency limit of the Kramers-Kronig relations [5]; in this case, the multiplication by *ω* before Eq. (1) is better motivated. Physically, the *f*-sum rule puts an important constraint on the absorption of electromagnetic radiation by a physical system. In this paper, we will consider how the *f*-sum rule applies to the transverse response of the electron gas.

The *f*-sum rule should apply to all response functions that satisfy Eq. (2) in the high frequency limit. Although the exact dielectric function for an electronic gas is unknown, Lindhard [6] provides an analytic approximate form that incorporates the basic physics of the problem. Lindhard’s dielectric function for the response of an electron gas to a longitudinal perturbation is widely discussed in textbooks [1–3]. In his paper, Lindhard also presents the dielectric function for the response of an electron gas to a transverse perturbation [6]. Little use has been made of the transverse response function, although, at least two articles report attempts to extend it to account for finite electron lifetime [7,8], and we used the function to discuss transverse response in the case of a fast electron traveling through the electron gas [9]. Recently, current-current susceptibility for the electron gas has been derived as part of the study of dynamic exchange-correlation potentials [10,11]. The current-current susceptibility *χ_JJ_* is very closely related to the dielectric function (including both its transverse and longitudinal parts); specifically Eq. (4.165) of Pines and Nozières [l] is (with a slight change of notation)

$$\overset{↔}{\varepsilon }(\vec{q},\omega )=1-\frac{\omega_{p}^{2}}{\omega^{2}}-\frac{4\pi e^{2}}{\omega^{2}}{\overset{↔}{\chi }}_{JJ}(\vec{q},\omega )$$ (5)

where $\overset{↔}{\varepsilon }$ is the dielectric tensor, ω is the frequency, and $\vec{q}$ is the wave vector. In a basis which diagonalizes $\overset{↔}{\varepsilon }(\vec{q},\omega )$, the dielectric tensor has two eigenvalues given by *ε* ^(^*^t^*^)^(*q*,*ω*) and *ε* ^(^*^l^*^)^(*q*,*ω*) which are the transverse and longitudinal dielectric functions, respectively [6,12]. The convention $\vec{q}=q\hat{q}$ is used, where *q* is the magnitude of the wave vector and $\hat{q}$ is a unit vector. Lindhard’s transverse function may be obtained from the results of Böhm, Conti, Nifosí, and Tosi [10,11] by the application of Eq. (5).

Lindhard gives the imaginary part of the transverse dielectric function as

Imε(t)(q,ω)=ωp2ω2×{3π4u(1−u2−z2)foru+z<13π32z[1−(u−z)2]2for|u−z|<1<u+z0for|u−z|>1 (6)

for *ω* > 0. (An overall sign error has been corrected; odd parity applies for negative *ω*.) Lindhard defines the dimensionless variables *z* = *q*/(2*k_F_*) and $u=\bar{\omega }/(k_{F}q)$, where $\bar{\omega }=m\omega /\hbar$, *ħ* is Planck’s constant, and *k_F_* is the Fermi wave vector. He gives the real part of the transverse dielectric function as

Reε(t)(q,ω)=1−ωp2ω2{38(z2+3u2+1)−332z[∑±[1−(z±u)2]2ln|z±u+1z±u−1|]}. (7)

The sum over ± is just the two-term sum of the formula with ± → + then ± → −. This function obeys Eq. (2) independently for each *q*. It is even stated in the literature that the transverse dielectric function *ε* ^(^*^t^*^)^ satisfies the *f*-sum rule of Eq. (4) [13].

Let’s check. The integrals are numerous but elementary, and the result is

∫−∞∞dωImε(t)(q,ω)ω=πωp2(58−38z2+316z(1−z2)2ln|1+z1−z|). (8)

A single functional form exists for all *z* (physically all *q*) despite the fact that Eq. (6) has various analytic forms. Equation (8) is $\pi \omega_{p}^{2}$ only for the limit of *q* → 0, whereas equality is expected for all *q* according to Martin [13]. The discrepancy is due to the existence of a pole term at *ω* = 0 [1,9]. Below, we rederive Lindhard’s transverse dielectric function with an emphasis on the second-order pole.

## 2. Transverse Response Revisited

We specialize the derivation of Adler [12] for the dielectric function of a periodic solid with band theory to the case of the electron gas. We find

$$\begin{array}{l} \overset{↔}{\varepsilon }(\vec{q},\Omega )=1-\frac{\omega_{p}^{2}}{\Omega^{2}}\\ \;+\frac{2\omega_{p}^{2}}{n\Omega^{2}}\sum_{\vec{k}}\frac{\left(\vec{k}+\vec{q}/2)⊗(\vec{k}+\vec{q}/2\right)}{\vec{q}\cdot \vec{k}+q^{2}/2}f_{\vec{k}}\\ \;+\frac{m^{2}\omega_{p}^{2}}{\hbar^{2}n}\sum_{\vec{k}\pm }\frac{\left(\vec{k}+\vec{q}/2)⊗(\vec{k}+\vec{q}/2\right)}{{\left(\vec{q}\cdot \vec{k}+q^{2}/2\right)}^{2}\left(\pm \bar{\Omega }+\vec{q}\cdot \vec{k}+q^{2}/2\right)}f_{\vec{k}}, \end{array}$$ (9)

where Ω is the frequency of the external perturbation including a small, positive imaginary part *iη* used in the “adiabatic turn-on” argument, i.e., Ω = *ω* + *iη*, *n* is the number density of the electron gas, and scalars are promoted with identity tensors as required. Here, $\overset{↔}{\varepsilon }$ is the dielectric tensor associated with the electric field derived from the vector potential, or equivalently, the dielectric function expressed in the Hamiltonian gauge which is defined by *ϕ* = 0. The scaled frequency is defined by $\bar{\Omega }=m\Omega /\hbar$. The $f_{\vec{k}}$ are the Fermi occupation numbers defined with Adler’s normalization convention, i.e., $f_{\vec{k}}=2/V$ for a fully occupied state, including a factor of 2 for spin degeneracy; here *V* is the system volume. Adler’s normalization convention leads to

$$\sum_{\vec{k}}f_{\vec{k}}\to \frac{2}{{\left(2\pi \right)}^{3}}\int d\vec{k},$$ (10)

as discussed, for example, in Ashcroft and Mermin [14]. For the electron gas, the energy of a state with momentum $\vec{k}$ is given by $E_{\vec{k}}=\hbar^{2}k^{2}/(2m)$, so

$$E_{\vec{k}+\vec{q}}-E_{\vec{k}}=\frac{\hbar^{2}}{m}\left(\vec{q}\cdot \vec{k}+\frac{q^{2}}{2}\right),$$ (11)

which appears in the derivation of Eq. (9). In deriving Eq. (9), we found that Eq. (5) needed to be corrected to

$$\overset{↔}{\varepsilon }(\vec{q},\omega )=1-\frac{\omega_{p}^{2}}{{\left(\omega +i\eta \right)}^{2}}-\frac{4\pi e^{2}}{{\left(\omega +i\eta \right)}^{2}}{\overset{↔}{\chi }}_{JJ}(\vec{q},\omega )$$ (12)

by the addition of *iη* to the two denominators.

Equation (9) includes both longitudinal and transverse response. These are uncoupled [12]. The longitudinal response may be obtained by forming $\hat{q}\cdot \overset{↔}{\varepsilon }(\vec{q},\omega )\cdot \hat{q}$. In this case the second and third terms of Eq. (9) cancel, and the first and final terms give rise to Lindhard’s longitudinal dielectric function, as we have verified in detail. (If the reader wishes to do so, we recommend forming the dimensionless variables κ = *q*/*k_F_* and $w_{\pm }=1/2\pm \bar{\omega }/q^{2}$ to minimize the algebra). Because the longitudinal response is usually derived from the scalar potential, this equivalence is a consequence of gauge invariance which Adler has proved in the more general context of periodic potentials of solids within band theory [12].

To find the transverse response, the form to ${\hat{q}}_{⊥}\cdot \overset{↔}{\varepsilon }(\vec{q},\omega )\cdot {\hat{q}}_{⊥}$ is required, where ${\hat{q}}_{⊥}$ is a unit vector orthogonal to $\hat{q}$. The real part of Lindhard’s transverse dielectric function is given by evaluating Eq. (9). (The integrals are reasonably elementary and very similar to the longitudinal case; we made use of a partial fractions expansion $\upsilon^{-2}{\left(\pm \bar{\omega }+\upsilon \right)}^{-1}=\pm \upsilon^{-2}{\bar{\omega }}^{-1}-\upsilon^{-1}{\bar{\omega }}^{-2}+{\bar{\omega }}^{-2}{\left(\pm \bar{\omega }+\upsilon \right)}^{-1}$ with υ = *qkx* + *q*^2^/2 and *x* = cos*θ* as well as $\int dt\;t^{n}\ln t={\left(n+1\right)}^{-1}t^{n+1}\ln t-{\left(n+1\right)}^{-1}t^{n+1}$. The imaginary part of Lindhard’s transverse dielectric function reproduced in Eq. (6) is given by just the fourth term of Eq. (9).

However, there remains an imaginary part associated with Ω^−2^, which is defined to be (*ω* + *iη*)^−2^. In the Appendix, we derive the relation

$$\frac{1}{{\left(\omega +i\eta \right)}^{2}}=P\frac{1}{\omega^{2}}-i\pi \delta (\omega )\frac{d}{d\omega }(\;)$$ (13)

by an argument which is analogous to the argument used to derive the well-known relation [2]

$$\frac{1}{\left(\omega +i\eta \right)}=P\frac{1}{\omega }-i\pi \delta (\omega ).$$ (14)

Here, P causes an integral to be evaluated as a principal value, and *δ* is the Dirac *δ* function.

Consider the dielectric function

$$\varepsilon (\omega )=1-\frac{\omega_{p}^{2}}{\Omega^{2}}.$$ (15)

Taking the imaginary part from Eq. (13), it is possible to form the *f*-sum rule integral of Eq. (4) as:

$$\int_{-\infty }^{\infty }d\omega (-\omega_{p}^{2})(-\pi )\delta (\omega )\frac{d}{d\omega }\omega =\pi \omega_{p}^{2}.$$ (16)

This example turns out to be the only relation that is not widely discussed which is needed to complete the discussion of the *f*-sum rule for Lindhard’s transverse dielectric function. Equation (15) is the first and second terms of Eq. (9), so Eq. (16) represents their contributions to the *f*-sum rule. (Of course, the first term, 1, has no imaginary part so has a zero contribution.) Equation (16) applies to the third terms of Eq. (9) as well, because the sum over $\vec{k}$ has no Ω dependence. Explicitly, the third term yields

$$\pi \omega_{p}^{2}\left(-\frac{5}{8}+\frac{3}{8}z^{2}-\frac{3}{16z}{\left(1-z^{2}\right)}^{2}\ln\left|\frac{1+z}{1-z}\right|\right)$$ (17)

which is just minus Eq. (8): the second term alone yields the expected answer of $\pi \omega_{p}^{2}$. Alternatively, the pole term may be found by grouping the second and third terms. Application of Eq. (13) to Eq. (9) yield the pole term [9]

$$\pi \omega_{p}^{2}\left(\frac{3}{8}+\frac{3}{8}z^{2}-\frac{3}{16z}{\left(1-z^{2}\right)}^{2}\ln\left|\frac{1+z}{1-z}\right|\right).$$ (18)

If this term is added to Eq. (8), the result is $\pi \omega_{p}^{2}$.

We emphasize that Eq. (8) and Eq. (18) were independently derived from Eq. (9) using Eq. (10), Eq. (13), and Eq. (14). We did not assume the value for the sum rule, but rather derived it. The discrete sum of Eq. (9) contributes a term at a pair of positive and negative frequencies, which are in general different for the different $\vec{k}$ and a zero frequency term for all $\vec{k}$. As the number of $\vec{k}$ points becomes larger, the strengths in a small frequency interval differ by an ever-increasing factor, which accounts for the singular contribution at zero frequency. In other words, the contribution at zero frequency is a finite multiple of the contribution from a finite interval at non-zero frequency. Such a situation cannot be accounted for within elementary integral calculus.

The traditional point of view is that the *f*-sum rule fails and the formula must be rewritten to include a pole term explicitly. Our point of view is that Eq. (18), when multiplied by the operator *δ* (*ω*)*d*()/*dω*, should be considered to be part of the imaginary part of *ε*^(t)^(*q*,*ω*), and that the *f*-sum rule is satisfied as written in Eq. (4). Because the imaginary parts of *ε*^(t)^(*q*,*ω*) arise from the same original formula, Eq. (9), and because the first-order poles and the second-order poles give analogous contributions, as shown in the Appendix, we see this accounting as more systematic, although less traditional.

## 3. Conclusions

The key requirements for the *f*-sum rule are causality, which implies there are no poles in the upper-half complex frequency plane, and the free-electron response at high frequencies. In the *f*-sum integrand, Im *ε* (*ω*)*ω*, the real axis gets contributions from poles which are located just below it. First-order poles in *ε* (*ω*) contribute for *ω* ≠ 0, but a second-order pole in *ε* (*ω*) contributes for *ω* = 0. The formula for contributions from such second-order poles is given by Eq. (13) which is analogous to the more familiar Eq. (14). It is somewhat unsettling that the principle “zero times anything is zero” does not apply to generalized functions as singular as those of Eq. (13). The traditional point of view is that the *f*-sum rule fails and a pole term is required. Our point of view is that the *f*-sum rule holds with a modified imaginary part of the dielectric function. In physics, the Dirac δ-function is widely used, but it is just one of a number of generalized functions which have been studied for over 50 years [15]. By introducing a higher generalized function to the imaginary part of the response function, causality and the *f*-sum rule become more tightly linked.

In the important special case of

$$\varepsilon (\omega )=1-\frac{\omega_{p}^{2}}{{\left(\omega +i\eta \right)}^{2}}$$ (19)

*only* the second-order pole contributes to the *f*-sum integral. The imaginary part of the dielectric function in Eq. (19) is rarely discussed because it is notationally awkward, without using notation such as that of Eq. (13), even though the *f*-sum rule for the inverse of Eq. (19) is widely discussed as a plasmon.

We appeal to authors and teachers to carefully distinguish between *ω* and Ω = *ω* + *iη*. The almost trivial difference between Eq. (5) and Eq. (12) took us several weeks to fully comprehend. One of the big ideas of physics is that if the assumptions and rules of inference are stated clearly enough, verifying a derivation is purely mechanical. This only works if the notation is crystal clear.

## 4. Appendix: Integral Relations for (*ω* + *iη*)^−1^ and (*ω* + *iη*)^−2^

Equation (14) is widely used in theoretical physics [2]. The imaginary part of this relation may be demonstrated as follows for any smooth bounded function *f* (*ω*):

A=limη→0Im∫−∞∞dωf(ω)ω+iη (A.1)

$$=-\underset{\eta \to 0}{\lim}\int_{-\infty }^{\infty }d\omega \frac{f(\omega )}{\omega^{2}+\eta^{2}}\eta$$ (A.2)

defining *A*. Let *u* = ω/η.

$$A=-\underset{\eta \to 0}{\lim}\int_{-\infty }^{\infty }du\frac{f(\eta u)}{1+u^{2}}$$ (A.3)

$$=-f(0)\int_{-\infty }^{\infty }du\frac{1}{1+u^{2}}$$ (A.4)

=−πf(0) (A.5)

establishing the result. The analogous derivation for (*ω* + *iη*)^−2^ is

B=limη→0Im∫−∞∞dωf(ω)(ω+iη)−2 (A.6)

$$=-2\underset{\eta \to 0}{\lim}\int_{-\infty }^{\infty }d\omega \frac{f(\omega )}{{\left(\omega^{2}+\eta^{2}\right)}^{2}}\omega \eta$$ (A.7)

$$=-2\underset{\eta \to 0}{\lim}\int_{-\infty }^{\infty }du\frac{f(\eta u)}{{\left(1+u^{2}\right)}^{2}}\frac{u}{\eta }$$ (A.8)

$$=-\underset{\eta \to 0}{\lim}\int_{-\infty }^{\infty }du\frac{u^{2}}{{\left(1+u^{2}\right)}^{2}}\frac{f(\eta u)-f(-\eta u)}{\eta u}$$ (A.9)

$$=-2\frac{df(\omega )}{d\omega }|{}_{\omega =0}\int_{-\infty }^{\infty }du\frac{u^{2}}{{\left(1+u^{2}\right)}^{2}}$$ (A.10)

$$=-\pi f^{\prime }(0).$$ (A.11)

Because the proof for the real part is nearly identical to the well-established (*ω* + *iη*)^−1^ case, it is omitted. This proves Eq. (13).
